# Risk factors for hemorrhage of brain arteriovenous malformation

**DOI:** 10.1111/cns.13200

**Published:** 2019-07-29

**Authors:** Sonali S. Shaligram, Ethan Winkler, Daniel Cooke, Hua Su

**Affiliations:** ^1^ Center for Cerebrovascular Research, Department of Anesthesia and Perioperative care University of California San Francisco California; ^2^ Department of Neurological Surgery University of California San Francisco California; ^3^ Department of Radiology University of California San Francisco California

**Keywords:** brain arteriovenous malformation, hemodynamic, intracranial hemorrhage, vascular endothelial growth factor, vascular integrity

## Abstract

Patients with brain arteriovenous malformation (bAVM) are at risk of intracranial hemorrhage (ICH). Overall, bAVM accounts for 25% of hemorrhagic strokes in adults <50 years of age. The treatment of unruptured bAVMs has become controversial, because the natural history of these patients may be less morbid than invasive therapies. Available treatments include observation, surgical resection, endovascular embolization, stereotactic radiosurgery, or combination thereof. Knowing the risk factors for bAVM hemorrhage is crucial for selecting appropriate therapeutic strategies. In this review, we discussed several biological risk factors, which may contribute to bAVM hemorrhage.

## 1 INTRODUCTION

Brain arteriovenous malformations (bAVMs) are abnormal vessels that are prone to rupture causing life‐threatening intracranial hemorrhage (ICH) and long‐term disability, especially in young adults.^1^ About 45% of bAVM cases present with hemorrhage. However, as many as 88% of bAVM patients are asymptomatic. Currently, there is no specific and safe medical therapy available to prevent bAVM hemorrhage. Risk factors for hemorrhage have not been consistent across longitudinal studies, primarily due to small sample sizes or selection biases of cases. A continuous identification of risk factors is important as the risk of hemorrhage can vary widely from 0.9% to 34.3%, depending on the number of overlapping risk factors carried by a patient.^2^ Furthermore, findings from a randomized trial of unruptured bAVM (ARUBA) showed that stroke and mortality were lower in unruptured bAVM patients randomized to conservative management than patients that received any interventional therapy.^3^ Therefore, there is an urgent need to identify risk factors to stratify patients who would benefit most from the treatment.

Molecular characterization of resected bAVM tissue has provided evidence for involvement of angiogenic and inflammatory pathways, but a complete understanding of pathogenic pathways and determinants of disease progression remain obscure. Recent works have shown that elevations of vascular endothelial growth factor (VEGF) or alterations in the vascular wall, such as a loss of pericytes, may contribute to bAVM rupture.^4^, ^5^, ^6^ Abnormally high blood flow through arterial‐venous shunting has also been suggested to contribute to bAVM rupture.

**Figure 1:**
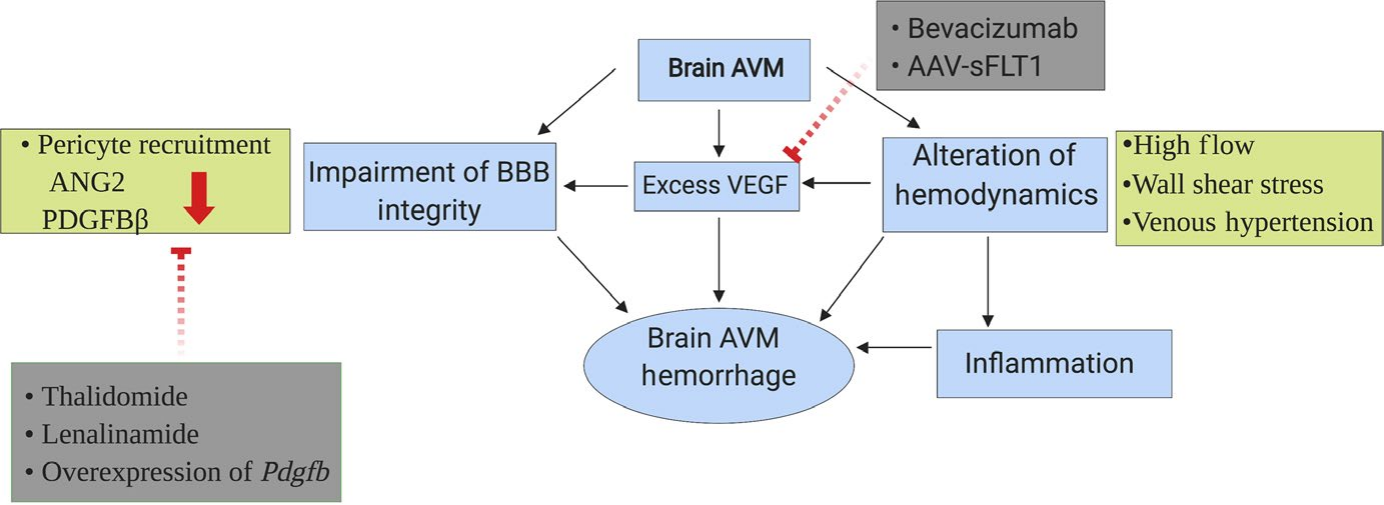
The risk factors for brain AVM hemorrhage. Brain AVMs have increased level of VEGF, reduced mural cell coverage, and altered hemodynamics. All of these increase the risk of brain AVM hemorrhage. Alteration in hemodynamics including high flow, increased wall shear stress, and VH can also induce inflammation, BBB leakage, and elevation of VEGF levels, which further increases the risk of hemorrhage. Thalidomide and lenalidomide treatment increase EC PDGFB production and pericyte recruitment. Overexpression of *Pdgfb* could be another therapeutic strategy to improve BBB integrity. Bevacizumab treatment and intravenous injection of AAV‐sFLT1 vectors that express the extracellular domain of VEGF receptor 1 blocks excess VEGF and inhibits the bAVM formation and progression

This review discussed the roles of VEGF, signaling pathways involved in vascular integrity, and hemodynamic changes in bAVM hemorrhage. We have also discussed current therapeutic options and the potential direction for development of specific medical treatment in the future (Figure 1).

## 2 ELEVATION OF VEGF LEVEL EXACERBATES BAVM HEMORRHAGE THROUGH IMPAIRMENT OF BLOOD‐BRAIN BARRIER (BBB) INTEGRITY

The VEGF family comprises in mammals five members: VEGF‐A, placenta growth factor (PGF), VEGF‐B, VEGF‐C, and VEGF‐D. The VEGF discussed in this review is VEGF‐A.

Vascular endothelial growth factor plays a crucial role in vascular remodeling and angiogenesis. VEGF receptor 1 (VEGFR‐1) and VEGF receptor 2 (VEGFR‐2) are the two main receptors mediating VEGF angiogenesis function. The downstream players of VEGFR‐2 signaling include (a) Ras/Raf/MEK, (b) PI3K‐AKT/PKB, and (c) p38/MAPK‐HSP27 pathways.^7^ Signaling through VEGFR‐2, VEGF increases vessel permeability in ischemic condition^8^ by disruption of endothelium tight junctions via downregulation of zonula occludens‐1 and disorganization of the actin cytoskeleton.^9^ Tight junction proteins, for example, occludin and claudin‐5, are also downregulated when VEGF level is correlated with BBB breakdown.^10^

### 2.1 Role of VEGF in bAVM hemorrhage

Brain AVM represents a rare but important source of neurological morbidity in young adults.^11^ The abnormally high levels of VEGF and VEGF receptors in endothelial cells (ECs) of surgically resected bAVM tissue have been reported.^12^, ^13^, ^14^ Animal models suggest that VEGF may contribute to the hemorrhagic tendency.^4^, ^15^ The high VEGF levels are associated with increased permeability of BBB and bAVM hemorrhage.^16^, ^17^, ^18^ The imbalanced expression of VEGF, angiopoietin‐1, and angiopoietin‐2 contributes to abnormal vascular remodeling causing impaired wall structure in bAVM vessels. We recently demonstrated a direct link between the elevated VEGF level and bAVM hemorrhage in a mouse bAVM model.^4^

Furthermore, it has been shown that plasma VEGF levels are elevated in bAVM patients.^19^ Contrastingly, a decreased serum VEGF level in patients with AVM was suggested to be through pooling of circulating VEGF within and around the nidus and concomitant depletion of systemic VEGF secretion due to negative feedback loop.^20^

### 2.2 Interplay of VEGF and hemodynamic changes

The abnormal vessels in human bAVMs are exposed to variable degrees of increased intraluminal flow and venous hypertension (VH). A positive correlation between VH and angiogenic activity was first proposed by Lawton et al.^21^ It has also been shown that VH upregulates the expression of VEGF and hypoxia‐inducible factor 1‐alpha (HIF‐1α).^22^, ^23^ Upregulation of the nuclear factor erythroid 2‐related factor 2 (Nrf2) and its downstream targets HIF‐1α and VEGF have been detected in human AVM samples and a rat VH model.^24^ Nrf2, a transcriptional factor, regulates antioxidant genes and influences angiogenesis. Therefore, the interplay between Nrf2 and VEGF might contribute to VH‐induced angiogenesis in bAVM pathogenesis.

Recently, we reported that creation of VH in mice with bAVMs caused severe hemorrhage in the bAVM lesions and high mortality.^4^ Therefore, elevation of VEGF induced by VH might be one of the responsible factors of bAVM hemorrhage.

### 2.3 Inhibition of VEGF

Vascular endothelial growth factor expression in the brain is high during embryonic development and is reduced in the adult brain. VEGF stimulation is necessary for induction of bAVM formation in adult mice.^25^, ^26^, ^27^ VEGF neutralization prevented and normalized AVM in an animal model for hereditary hemorrhagic telangiectasia 2 (HHT2), an autosomal‐dominant disorder characterized by telangiectasia and AVMs in multiple organs.^28^ Bevacizumab (an antihuman VEGF antibody) treatment inhibits the bAVM formation and progression.^29^ Furthermore, intravenous injection of an adeno‐associated viral vector expressing sFLT1 (the extracellular domain of VEGF receptor 1) attenuated the phenotype severity of bAVMs in mice.^30^

These data indicated that VEGF plays a crucial role in bAVM hemorrhage and can be a therapeutic target.

## 3 **ROLES OF PLATELET‐DERIVED GROWTH FACTOR‐B** (**PDGFB) SIGNALING AND PERICYTES IN BAVM HEMORRHAGE**

Brain ECs form a one cell thick lining of the vascular lumen. Adjacent cells are tightly connected via tight and adherens junctional proteins, including claudins, occludins, and vascular endothelial cadherin, and form the BBB.^31^, ^32^ This EC barrier excludes large, nonlipophilic molecules (>40 Da), such as circulating bloodborne cells or plasma proteins, from the brain unless specific transport proteins are present.^31^, ^32^, ^33^, ^34^ Although the ECs are the anatomic site of the BBB, much of the structural integrity of the vascular wall comes from extracellular matrix and other surrounding cells—such as vascular smooth muscle cells or pericytes. ECs are embedded within a vascular basement and serves as a vital structural scaffolding comprised of laminins, fibronectins, collagens, and heparin sulfate proteoglycans.^35^ Differential expression of basement membranes, for example, perlecan, or adhesion molecules, such as integrins, responsible for anchoring vascular cells to the basement membrane has been reported and associated with AVM formation and rupture.^36^, ^37^, ^38^

The identity of the mural cells (vascular smooth muscle cells and pericytes) is dependent on the location along the arterial‐venous axis.^39^ In larger arteries, concentric rings of vascular smooth muscle cells are formed. As they branch into more distal arterioles, vascular smooth muscle cells become less numerous and no longer form concentric rings.^40^ In capillaries, vascular smooth muscle cells are replaced by the pericyte as the principle cell component of the vascular wall, which extend finger‐like cell processes covering much of the ECs.^33^, ^34^, ^41^ Crosstalk between mural cells promotes EC barrier properties.^32^, ^42^, ^43^, ^44^ Mural cells express both contractile proteins and proteins of the extracellular matrix, which help regulate vascular diameter.^45^, ^46^, ^47^, ^48^, ^49^ Loss of vascular smooth muscle cells and pericytes are associated with ectasia, aneurysm formation, and either leakage or rupture of arteries and capillaries, respectively.^33^, ^42^, ^43^, ^44^, ^47^, ^50^, ^51^, ^52^

### 3.1 PDGFB/PDGF Receptor β (PDGFRβ) Signaling Pathway

One important pathway for EC‐mural cell crosstalk is the PDGFB/PDGFRβ pathway.^32^, ^33^, ^41^ PDGFB is secreted from the endothelium as a disulfide‐linked homodimer and retained within the extracellular matrix as the result of electrostatic interactions.^53^, ^54^ This creates a steep perivascular concentration gradient of PDGF‐BB shown to be essential for the recruitment of mural cells—including migration, attachment and proliferation.^42^, ^54^, ^55^ Both pericytes and vascular smooth muscle cells express PDGFRβ—a tyrosine kinase receptor.^35^ PDGF‐BB binding to PDGFRβ triggers receptor dimerization, autophosphorylation, and activation of multiple downstream signal transduction pathways—including multiple Src homology 2 binding proteins, GTPase activating protein, SH2 tyrosine phosphatase, and phospholipase Cγ0.^32^ Deletion or genetic manipulation of *Pdgfb* or *Pdgfrβ* results in deficiency of vascular smooth muscle cells and pericytes.^35^, ^42^, ^43^, ^44^, ^56^, ^57^ A common consequence of reductions of pericytes or vascular smooth muscle is breakdown of the BBB, leakage of circulating plasma proteins into the brain and hemorrhage,^35^, ^42^, ^43^, ^44^, ^56^, ^57^ and homozygous deletion of *Pdgfb* or *Pdgfrβ* in rodents results *in utero* death due to widespread hemorrhage.^58^

### 3.2 BBB integrity and mural cell recruitment in bAVM pathogenesis

Abnormal expression of PGDF‐B and PDGFRβ has been described in bAVMs in rodent models and patients.^6^, ^59^, ^60^ Recent works showed that both human and mouse bAVM vessels have less mural cell coverage compared to normal brain vessels,^5^, ^6^, ^59^ suggesting an abnormal vascular remodeling in bAVMs. The number of pericytes is inversely correlated with the degree of overt symptomatic hemorrhage or clinically occult microhemorrhage.^5^, ^59^ However, vascular smooth muscle cells have yet to be fully characterized in human AVMs, and the functional consequences of other described abnormalities—such as cytoskeleton and contractile proteins—remain unclear.^61^, ^62^, ^63^

Despite these uncertainties, vascular smooth muscle cells and pericytes are an emerging therapeutic target through pharmacological manipulation of PDGFB.^6^, ^41^, ^64^ HHT patients are characterized by systemic mucosal capillary dilations, which are prone to bleeding resulting in epistaxis or gastrointestinal bleeding. Approximately, 5%‐23% of patients with HHT develop brain vascular malformations—including AVMs.^65^, ^66^ In HHT patients, thalidomide treatment was shown to reduce epistaxis. Increases in EC PDGFB production and enhanced pericyte recruitment were demonstrated to exert thalidomide's vascular stabilizing effect in *Eng^+/−^* rodents.^64^ More recently, treatment with thalidomide or its less toxic analog—lenalidomide—was shown to increase recruitment of both pericytes and vascular smooth muscle cells in mouse bAVMs.^6^ Enhanced mural recruitment was associated with reductions in vascular dysplasia and hemorrhage. Mechanistic experiments confirmed that this effect was the result of increased EC *Pdgfb* expression. Overexpression of *Pdgfb* recapitulated the therapeutic benefit of thalidomide.^6^ These studies provided first proof‐of‐principle evidence that vascular smooth muscle and pericytes may represent novel therapeutic targets in bAVMs. Whether these results may be translated into human patients to stabilize bAVMs remains to be seen.

## 4 ALTERATION OF HEMODYNAMICS EXACERBATES BAVM HEMORRHAGE

On functional level, an AVM is a collection of vessels that transmit a disproportionately higher per unit volume of blood relative to its surrounding vasculature. This phenomenon is important in understanding the pathophysiology of AVMs in that these nonphysiological hemodynamics manifest forces that can affect the molecular and structural composition of a vessel wall. We described flow in dimensions of pressure, velocity, and organization (eg, laminar or turbulent) as functions of time, geometry, and surface area. The collective study of these metrics, namely computational fluid dynamics (CFD), is expansive and complex. When applied in relatively simple biological models (eg, flow chamber), meaningful relationships between cell biology and hemodynamics crystalized. Brain AVMs, however, are considerably more complicated and challenging to study.^67^

### 4.1 Hemodynamics in bAVMs

A key aspect in discussing the role of fluid dynamics in AVMs is to understand its relevance to stroke risk as part of disease natural history or therapeutic intervention. Currently, there is no reliable method to predict AVM‐related ICH due to inconsistency in clinical and angioarchitectural risk factors across series. More flow metrics might improve identification of patients at risk. Alternatively, we also have uncertainty of posttherapeutic ICH, such as after microsurgical, embolization, or gamma knife treatments.^68^ Demonstrating flow parameters preintervention that elevate posttreatment risk could help direct patients to alternative therapies and/or different postsurgical management.^69^, ^70^, ^71^, ^72^, ^73^ Research into both of these clinical scenarios has enlisted hemodynamic metrics to help explain such hemorrhagic episodes with varying degrees of success.

### 4.2 Flow in brain hemorrhage

With the exception of aneurysm‐related subarachnoid hemorrhage, the precise vascular locus of AVM‐related ICH is unknown. There is varying evidence for potential location(s), which could be at an arterial, nidal, and/or venous sites. Knowing precisely which vascular compartment was at risk would be helpful in targeting fluid dynamics. Even in the absence of this clarity, much has been learned on how hemodynamics may affect ICH risk. There are many studies characterizing fluid dynamics within aneurysms^74^, ^75^, ^76^ with or without ruptured. However, these studies are inconsistent in defining which and in what direction fluid dynamic parameters lead to aneurysm rupture. Many groups have focused on wall shear stress and its association with aneurysmal wall inflammation.^77^, ^78^, ^79^, ^80^ However, there are few longitudinal studies detailing this question and lack of consistency between groups on how to generate such CFD values. There is evidence that high‐flow arteries carry a greater likelihood of aneurysm formation than those anatomically matched, low flow vessels. For example, Shakur et al, using MRA‐based flow methods, noted increased flow rates and relative wall shear stress in AVM arterial afferents harboring aneurysms than those that did not.^81^ However, there is again limited information about which hemodynamic parameters can predict when and where an AVM‐related aneurysm may form. Ultimately, 15%‐50% of AVMs have aneurysms.^82^, ^83^, ^84^, ^85^, ^86^, ^87^, ^88^ Whether the presence of aneurysms is a risk factor of ICH is controversy.^82^, ^83^, ^86^, ^89^, ^90^ As such, more effort in the evaluation of the role of hemodynamics on AVM natural history has gone into its relation to the nidus rather than any specific prenidal arterial segment.

AV shunting is pathological not only in that it reduces or even eliminates the physiological exchange of blood gases, nutrients, and waste products a tissue bed requires, but it also exposes veins to supraphysiological pressures. Veins of the brain are thinned walled, valveless vessels designed for passive return of blood to the cardiopulmonary circuit. In the setting of an AV shunt, the vein(s) walls will thicken in response to the increased pressure. Over time such neointimal hyperplasia can cause the vein(s) to narrow and/or occlude, evidenced commonly in the surgical dialysis arteriovenous fistula population as well as in AVM patients.^91^, ^92^, ^93^, ^94^, ^95^, ^96^ Based on this rationale, many groups have thus studied flow parameters to the nidus to determine relationships with ICH and other clinical and treatment outcomes. Bolstering this rationale, even if inconsistent, is an observation that smaller AVMs with a single and/or stenosed draining vein are more likely to have hemorrhagic clinical presentation.^97^

### 4.3 Methods for flow assessment

Technically, capturing such flow information has been difficult over the past 40 years. Qualitative assessments of flow are helpful, although the reproducibility and nuance of such measures are poor. For quantitative methods, radiotracers and transcranial ultrasound were used initially,^98^, ^99^ the use of MR has become more common. MR is significantly more sensitive, accurate, and comprehensive tool in capturing the flow metrics in feeding arteries and draining veins. There are a few studies discussed contradictions in the use of MR to analyze the role of hemodynamics in bAVM hemorrhage. For example, Illies et al, in 72 patients undergoing 4D MRA, noted no association between historical AVM ICH risk factors and hemodynamic metrics save that prior ICH was associated with increased transit time by 2.4 seconds (95% CI, 1.2‐3.6 seconds, *P* < 0.001).^100^ Similarly, Shakur et al,^101^ using quantitative MRA, noted no relationship between indices of arterial afferent pulsatility or resistance and historical angioarchitectural ICH risk factors. Conversely, Raoult et al, using 4D MRA, noted that the draining‐vein‐to‐arterial‐feeder time‐to‐peak ratio was significantly lower in the hemorrhagic compared with nonhemorrhagic patients (1.50 vs 2.1; *P* = 0.001),^102^ and Todaka et al^103^ demonstrated a significant difference in the mean number of draining veins (1.50 vs 2.3; *P* = 0.006) and the mean transit times (MTT) of the feeding artery (1.10 vs 1.62; *P* = 0.03). There are additional series further supporting both positions that MR‐based flow metrics do and do not^104^ help predict ICH events.

Despite these efforts, MR‐based methods are more complicated^105^ and require experts during MR scanning to focus regions of interest and provide the real‐time feedback during catheter angiography. In the setting of endovascular treatment, angiography is always performed providing the most sensitive and specific information of vascular anatomy and blood flow, though the efforts to quantitate such flow have been limited. Norris et al^106^ used digital subtraction angiograph (DSA) to measure arterial time‐to‐peak and nidal MTT within 31 bAVM patients noting an association between prolongation of time to peak (TTP) and shorter MTT and hemorrhagic presentation. This is congruent with some of the above MR‐based studies implicate increased upstream resistance, nidal, and/or venous as an ICH risk factor. More recently, groups have used parametric color coding, a technique that instantaneously converts a 2D X‐ray angiogram into a color‐map image where the time dimension is color‐encoded.^107^ The power of the technique is its simplicity and immediacy to the operator. The outputs enable production of time‐contrast density curves that then can be visually inspected and quantified. For example, Chen et al^108^ noted that patients with clinically occult nidal microhemorrhage demonstrated shorter nidal MTT. Some of these studies have been helpful as it relates to determine intranidal flow and detailed hemodynamic measurements within any given nidal vessel can be difficult given their complex anatomy. This technique is limited in that it is motion‐sensitive, and vessels of interest may overlap other vascular anatomy reducing signal clarity. Most importantly, it does not provide a true measure of flow as MR does. There is evidence of agreement between the modalities on this measure; for example, Brunozzi et al and Shakur et al reported correlation between MR‐based flow analysis and DSA‐based contrast time‐density curves, with the former also noting that ICH presentation was more common in those with decreased venous transit times and the latter decreased MTT and seizure presentation.^109^, ^110^ These studies are promising, and more recent work has expanded the technology to 3D datasets^111^ and treatment assesments.^73^ More work needs to be done validating the parametric color‐coding method in addition to bAVM hemodynamics and natural history in general.

## 5 CURRENT TREATMENTS AND FUTURE DIRECTIONS/DEVELOPMENTS PROSPECTION

Despite recent advances in bAVM molecular biology, no established medical therapy presently exists. Available treatment options include observation, surgical resection, endovascular embolization, stereotactic radiosurgery, or combination thereof.^112^ The advantages and disadvantages of these therapies are summarized in Table 1. Recent completed two randomized clinical trials or prospective registries suggested that risks of treatment may outweigh risks of future hemorrhage and favor observation.^3^, ^113^ The ARUBA is a randomized trial aimed to compare the risk of death and symptomatic stroke in patients with an unruptured bAVM who were allocated to either medical management alone or medical management with interventional therapy. The data obtained from ARUBA trial showed that medical management alone is superior to medical management with interventional therapy for the prevention of death or stroke in patients with unrupture bAVMs.^3^ The Scottish Audit of Intracranial Vascular Malformations (SAIVM) compared the long‐term outcomes of conservative management vs intervention for unruptured bAVM. This study found that among patients aged 16 years or older, the conservative management was associated with better clinical outcomes for up to 12 years compared with intervention.^113^ Numerous centers have since reported superior safety profiles with treatment.^114^, ^115^, ^116^, ^117^, ^118^, ^119^, ^120^ The optimal treatment remains controversial.

**Table 1:** The advantages and disadvantages of current bAVM treatments

Treatment	Advantages	Disadvantages	Usage
Microsurgery	‐ High complete angiographic exclusion (CAE) rates (>90%) for lower grade lesions ‐ Immediate effect (better for hemorrhagic lesions) ‐ Tissue collection for genetic analysis	‐ Invasive (morbidity) ‐ Operator dependent	‐ Multiple, prospective, and retrospective case series
Radiosurgery	‐ Less invasive ‐ Decreased morbidity for higher grade lesions ‐ Less operator dependent ‐ Reduced cost	‐ Delayed effect ‐ Lower rates of CAE (relative microsurgery) ‐ Requires multiple sessions ‐ Adverse radiation associated events	‐ Multiple, prospective, and retrospective case series
Embolization	‐ Less invasive ‐ Immediate effect (better for hemorrhagic lesions)	‐ Operator dependent ‐ Heterogeneous materials and technical details ‐ Lower rates of CAE (relative microsurgery) ‐ May require multiple sessions ‐ Cost	‐ Few, small prospective and retrospective case series
Combination therapy (M + E)	‐ For higher grade (SM > 2) lesions, postembolization reduced operative blood loss ‐ Targeted embolization of high‐risk features (ie aneurysms) ‐ Higher CAE for higher grade (SM > 2) lesions, relative to monotherapy ‐ More immediate effect	‐ Cost ‐ Increased morbidity ‐ Less standardized	‐ Few, small retrospective series
Combination therapy (M + R)	‐ For higher grade (SM > 2) lesions, postradiosurgery reduced nidal volume to facilitate resection ‐ Targeted fibrosis of eloquent regions (eg, brainstem) prior to resection ‐ Higher CAE for higher grade (SM > 2) lesions, relative to monotherapy	‐ Delayed effect ‐ Cost ‐ Adverse radiation associated events	‐ Few, small retrospective series
Combination therapy (R + E)	‐ For higher grade (SM > 2) lesions, postradiosurgery reduced nidal volume to facilitate embolization ‐ Targeted fibrosis of high‐risk regions (eg, brainstem) prior to embolization ‐ Targeted embolization of high‐risk features (ie aneurysms)	‐ Delayed effect ‐ Cost ‐ Heterogeneous effects depending order of treatment ‐ Less standardized ‐ Adverse radiation associated events	‐ Few, small retrospective series
Combination therapy (R + E + M)	‐ For higher grade (SM > 3) lesions, postembolization reduced operative blood loss ‐ Targeted embolization of high‐risk features (ie aneurysms) ‐ Higher CAE for higher grade (SM > 2) lesions, relative to monotherapy ‐ More immediate effect	‐ Cost ‐ Delayed effect ‐ Cost ‐ Heterogeneous effects depending order of treatment	‐ Few, small retrospective series

### 5.1 Neurosurgical approaches

For AVMs located near the brain surface or easily accessible through open neurosurgical approaches, surgery has the highest rates of complete cure with an acceptable safety profile.^121^ Nuances for the surgical approach for bAVMs have been reviewed elsewhere.^121^ Candidacy for surgery is assigned by grading rubrics—such as the Spetzler‐Martin and Lawton‐Young supplementary scores.^122^, ^123^, ^124^ These scoring systems took into account size or morphology of the nidus, pattern of venous drainage, lesion location, and whether the location fulfills an eloquent brain function, age, and rupture status. A combined score of ≤6 are generally thought to have a risk‐benefit profile favorable for surgery, whereas those ≥7 are often evaluated for other therapies.^121^

### 5.2 Stereotactic radiosurgery

Stereotactic radiosurgery is another less, invasive treatment strategy in which targeted radiation induces vascular damage and gradually leads to occlusion of the AVM.^121^ Radiation results in endothelial degeneration and vascular smooth muscle proliferation, which occludes or compresses the vascular lumen.^125^, ^126^ Radiation also decreases circulating levels of multiple proangiogenic factors within three months, including VEGF, TGF‐β, angiopoietin‐2, and basic fibroblast growth factor.^127^ However, radiation takes several years to have its effect during which bleeding may occur and also effects the adjacent brain resulting in nonspecific radiation induced changes or rarely radiation induced malignancies or other vascular malformations which may cause neurological symptoms.^121^

### 5.3 Endovascular embolization

Endovascular embolization is an alternative treatment method, though is largely used as a presurgical adjuvant to reduce microsurgical risk. The evidence supporting the use of embolization of bAVMs, whether as a primary treatment or adjuvant, is limited and controversial. The rarity and heterogeneity of bAVMs, along with the variation in embolization techniques and materials, make formal study of its role in treatment difficult to assess. Nonetheless, there are individual and collective series that provide guidance for how to best use embolization. In the most common scenario, where embolization is performed prior to microsurgery, there are a few series noting its impact on reducing blood loss relative to comparatively surgical grade lesions.^128^, ^129^, ^130^, ^131^, ^132^, ^133^ More recently, however, Donzelli et al noted no such effect, with their analysis also including operative time and the use of microclips as representative variables of surgical complexity. It is noteworthy that the majority of embolizations were performed using n‐cyanobutyl acrylate (nBCA) and there is evidence that ethylene vinyl alcohol (EVOH) copolymer may be a more effective material for nidal penetration, which may in turn have impacted the results.

Alternative strategies for embolization include targeted embolization of discrete high‐risk features (eg, aneurysm). Alexander et al demonstrated that embolization of nidal or perinidal aneurysmal AVM bleeding sites can reduce rebleeding within the first year following initial hemorrhage when compared to matched patients not undergoing such treatment.^134^ Such a practice has been advocated by others too.^128^ As it relates to attempted curative embolization, using transarterial or transvenous methods, there are a collection of small series noting high angiographic cure rates for bAVMs using embolization alone, though many of these series note higher rates of clinical complication relative to microsurgery and with limited clinical outcomes or long‐term follow‐up data.^135^ Finally, as it relates to the use of adjunctive embolization in radiosurgical practice, there is evidence of reduced obliteration rates relative to nonembolized lesions undergoing radiosurgery.^136^

### 5.4 Future development

As mentioned, there is no medical therapy for bAVMs at this time, although with the expansion of molecular understanding of these lesions, and their various genetic subgroups, treatments may be available. The use of bevacizumab, mitogen‐activated protein kinase enzyme (MEK) inhibitors, rapamycin, and thalidomide has all been used with varying degrees of efficacy for patients with non‐CNS AVMs. For example, the anti‐VEGF monoclonal antibody, bevacizumab, has been used to treat HHT‐related AVMs 137, 138 and radiation‐related injuries.^139^ Anti‐angiogenic drugs such as thalidomide have also been used to reduce bleeding of AVMs in HHT patients and GI syndrome patients.^140^, ^141^ In addition, other studies identified the role of RAS/MAPK and PI3K/mTOR pathways, which are involved in regulation of vascular growth and organization, in pathogenesis of AVMs. It has been shown that delivery of MEK inhibitors to AVM ECs may lead to reduced ERK activity and decreased vessel abnormalities,^142^, ^143^ while delivery of rapamycin, an mTOR inhibitor, has demonstrated positive effects on patients with varying vascular anomalies including AVMs.^144^, ^145^

Since the most devastating symptom of bAVM is ICH, unlike cancer‐related chemotherapy that aims to shrink abnormal tumor tissue, the concept for the treatment of bAVM should be to stabilize vascular tissue and thereby decrease the risk of spontaneous ICH. Therefore, all of aforementioned pathways and agents might be used to develop strategies to reduce bAVM size or hemorrhage.

## CONFLICT OF INTEREST

The authors have declared that no conflict of interest exists.
